# First high-resolution synchrotron X-ray study of icosahedrite, a natural quasicrystal from the Khatyrka meteorite

**DOI:** 10.1107/S2052252525006141

**Published:** 2025-07-22

**Authors:** Y. Ishii

**Affiliations:** a Department of Physics Chuo University Kasuga Tokyo112-8551 Japan

**Keywords:** quasicrystals, icoasahedrite, phasons, synchrotron radiation, Khatyrka meteorite

## Abstract

Quasicrystals are long-range-ordered materials with rotational symmetry incompatible with periodicity. Takakura *et al.* [(2025). *IUCrJ***12**, 435–443] present a study of a single grain of icosahedrite, the first natural icosahedral quasicrystal, which was found in a meteorite in 2009. Through detailed analysis of the diffraction peaks, they conclude that natural AlCuFe is an icosahedral quasicrystal superimposed by a phasonic modulation along the fivefold directions, which is similar to that observed in the synthetic quasicrystal. Based on knowledge of the synthesis and phase stability of icosahedral AlCuFe, they discuss the formation of icosahedrite in the meteorite.

Quasicrystals (QCs) are long-range-ordered materials with rotational symmetry incompatible with periodicity (Levine & Steinhardt, 1984 ▸). QCs were first discovered in rapidly quenched Al–Mn alloys with a diffraction pattern of icosahedral symmetry (Shechtman *et al.*, 1984 ▸) and a thermodynamically stable QC, Al_65_Cu_20_Fe_15_, synthesized by a conventional method of solidification, was discovered a few years later (Tsai *et al.*, 1987 ▸). This proved that a QC is not a non-equilibrium state of matter like an amorphous solid, but is an equilibrium phase that can be identified in a phase diagram. Since then, QCs have been obtained for more than 100 compounds and are now recognized as universal phases of matter. In 2009, the first natural icosahedral QC, Al_63_Cu_24_Fe_13_, was discovered in a mineral from the Koryak Mountains in Russia and named icosahedrite (Bindi *et al.*, 2009 ▸). Afterwards it was argued that the sample was of extraterrestrial origin, that is, part of a meteorite, likely formed in the early solar system (Bindi *et al.*, 2012 ▸).

Aperiodic crystals like QCs are characterized by a diffraction pattern with sharp Bragg spots that can be indexed with more than three integers and described as periodic structures in a superspace of more than three dimensions. Aperiodicity in QCs arises from competing incommensurate length scales inherent in the non-crystalline rotational symmetry. If one describes a periodic structure in a *d*-dimensional superspace (*d* = 6 for the icosahedral case) as a superposition of fundamental density waves with *d*-dimensional wavevectors **Q**^(*i*)^ (*i* = 1,…, *d*), the phase of each density wave φ^(*i*)^ (*i* = 1,…, *d*) results in modification of the QC structure. These phase degrees of freedom are conveniently represented with the displacement vector in the *d*-dimensional superspace **U** as
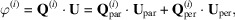
where **A** · **B** is a scalar product of vectors. One can divide the *d*-dimensional space into the three-dimensional physical space and the (*d*−3)-dimensional space perpendicular to the physical one, which is called the perpendicular space. Then 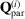 and **U**_par_ are the physical-space components of the wavevector **Q**^(*i*)^ and the displacement **U**, and 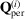 and **U**_per_ are their counterparts in the perpendicular space. **U**_par_ and **U**_per_ are called phonon and phason variables.

Various structural characteristics in real QCs are understood in terms of spatial variation of the phason variables (Fujiwara & Ishii, 2008 ▸). For example, if the phason variables depend linearly on the physical-space coordinates **r** as
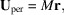
where *M* is a constant matrix called the phason strain tensor, the Bragg peaks shift from ideal positions by 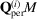. By choosing the phason strain appropriately, one can generate a periodic structure called an approximant (Ishii, 1989 ▸), which is related to a crystalline phase found near the QC composition in a phase diagram. If the phason strain is randomly frozen, the diffraction peaks acquire peak broadening proportional to 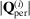. To characterize the quality of samples, the diffraction peak widths are usually checked as a function of 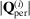 (Lubensky *et al.*, 1986 ▸). Even in high-quality samples of QCs, the long-wavelength phason fluctuations are observed as diffuse scattering (de Boissieu *et al.*, 1995 ▸), suggesting softening of the phason elastic constants (Widom, 1991 ▸; Ishii, 1992 ▸). Furthermore, the softening causes phasonic modulation where the phason variables undulate sinusoidally with the wavevector **q**_par_ as
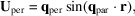
leading to the Bragg peaks being accompanied by satellite scattering at 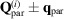 (Menguy *et al.*, 1993 ▸). This modulation is what is in fact found in the sample studied by Takakura *et al.* (2025 ▸).

Structural characterization of natural QCs has usually been based on electron diffraction. The paper by Takakura *et al.* presents the first results of a high-resolution synchrotron X-ray study of a single grain of icosahedrite. To clarify the structural characteristics, the authors stress that the high resolution is mandatory in order to look for possible modulations or microcrystalline states, whereas the high flux is important to measure precisely any diffuse scattering originating from phason fluctuations. Through detailed analysis of the diffraction peaks (see Fig. 1 ▸), the authors conclude that the natural AlCuFe is an icosahedral QC superimposed by a phasonic modulation along the fivefold directions with a wavelength of 20 nm, suggesting a phasonic instability along the fivefold direction in icosahedral QCs. A similar modulation was observed in synthetic AlCuFe as an intermediate phase between the high-temperature quasicrystalline state and the low-temperature rhombohedral crystalline state (Menguy *et al.*, 1993 ▸). To compare the icosahedrite crystal quality with that of the samples obtained in the laboratory by slow cooling from the melt, the mosaic spread of the single-crystal data and the residual phason strain that causes the Bragg peak broadening proportional to |**Q**_per_| are checked. It is remarkable that the quality of the natural QC is just as good as the best-quality synthetic QC. Based on the known facts about the synthesis and phase stability of icosahedral AlCuFe, the authors propose a possible scenario for the formation process in the meteorite, in which, under shock and at high temperature, the QC formed and grew as a single crystal in the range 850–800°C, followed by relatively slow cooling to about 600°C, which seems rather moderate compared with the earlier suggestion that icosahedrite formed under extreme impact conditions in space, at a temperature exceeding 1400 K and pressures above 5 GPa (Bindi *et al.*, 2009 ▸).

The meteorite sample consists of many small grains of crystals probably because of the large inhomogeneities in terms of pressure, temperature, thermal history and chemical composition in the formation process. This complexity makes the structural characterization of the natural QC more difficult. Use of an intense synchrotron light source and the state-of-the-art experimental methods developed for QC research succeeded in clarifying the character of the natural QC.

The discovery of QCs brought innovations in material science and solid-state physics. It is extraordinary that the natural QC found in a mineral or a meteorite is nearly identical to the synthetic icosahedral QC. This is another piece of evidence for QCs being a universal phase of matter and well defined equilibrium phases that can be identified in a phase diagram. It is also exciting that detailed information and knowledge of the synthesis of a QC obtained in the laboratory shed light on the formation of a QC in outer space. I hope QCs will continue to provide us with ‘something exciting to show you’ (Steinhardt, 2019 ▸).

## Figures and Tables

**Figure 1:**
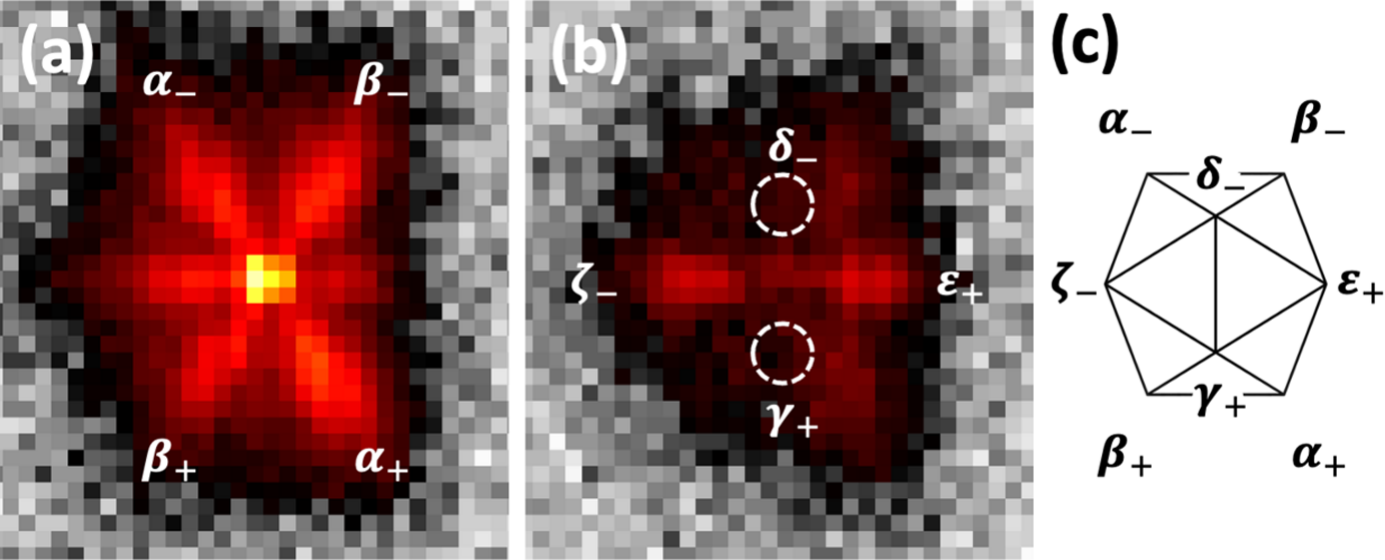
Intensity distribution (*a*) around the twofold 8/12 main Bragg reflection in icosahedrite and (*b*) at a slice just above Δ = 0.298 nm^−1^. (*c*) Layout of the satellite reflections. From Takakura *et al.* (2025 ▸).
